# Automatic imitation of human and computer-generated vocal stimuli

**DOI:** 10.3758/s13423-022-02218-6

**Published:** 2022-11-28

**Authors:** Hannah Wilt, Yuchunzi Wu, Antony Trotter, Patti Adank

**Affiliations:** 1 Department of Speech, Hearing and Phonetic Sciences, University College London, London, WC1N 1PF UK; 2 Department of Neural and Cognitive Sciences, New York University Shanghai, Shanghai, China; 3 NYU-ECNU Institute of Brain and Cognitive Sciences at New York University Shanghai, Shanghai, China; 4 Institute of Psychiatry, Psychology & Neuroscience, King’s College London, London, UK

**Keywords:** Imitation, Speech perception, Speech production, Vocal

## Abstract

Observing someone perform an action automatically activates neural substrates associated with executing that action. This covert response, or *automatic imitation*, is measured behaviourally using the stimulus–response compatibility (SRC) task. In an SRC task, participants are presented with compatible and incompatible response–distractor pairings (e.g., an instruction to say “ba” paired with an audio recording of “da” as an example of an incompatible trial). Automatic imitation is measured as the difference in response times (RT) or accuracy between incompatible and compatible trials. Larger automatic imitation effects have been interpreted as a larger covert imitation response. Past results suggest that an action’s biological status affects automatic imitation: Human-produced manual actions show enhanced automatic imitation effects compared with computer-generated actions. Per the integrated theory for language comprehension and production, action observation triggers a simulation process to recognize and interpret observed speech actions involving covert imitation. Human-generated actions are predicted to result in increased automatic imitation because the simulation process is predicted to engage more for actions produced by a speaker who is more similar to the listener. We conducted an online SRC task that presented participants with human and computer-generated speech stimuli to test this prediction. Participants responded faster to compatible than incompatible trials, showing an overall automatic imitation effect. Yet the human-generated and computer-generated vocal stimuli evoked similar automatic imitation effects. These results suggest that computer-generated speech stimuli evoke the same covert imitative response as human stimuli, thus rejecting predictions from the integrated theory of language comprehension and production.

**Supplementary Information:**

The online version contains supplementary material available at 10.3758/s13423-022-02218-6.

## Introduction

Observing others’ manual or vocal actions activates neural mechanisms required to perform that action (Buccino et al., 2004; Fadiga et al., 1998). For vocal actions, this type of covert—or automatic—imitation occurs whenever we hear and/or see someone speak and involves activation of speech production mechanisms and associated neural substrates (Fadiga et al., 2002; Nuttall et al., 2016; Watkins et al., 2003). Covert imitation is generally referred to as *automatic imitation* and is measured behaviourally using the stimulus–response compatibility (SRC) paradigm for manual and for vocal actions (Brass et al., 2000; Cracco et al., 2018; Heyes, 2011; Kerzel & Bekkering, 2000, Jarick & Jones, 2009). In a manual SRC task (Brass et al., 2000), participants are instructed to perform a manual action following a prompt (e.g., lift index finger for “1” and middle finger for “2”). The prompt is presented superimposed on a distractor image or video of a hand lifting the index or middle finger. When the prompt is presented in the presence of a compatible distractor (“1” with a video of a lifting index finger), participants are faster to perform the correct response than when the prompt is presented together with an incompatible distractor (“1” with a lifting middle finger video). For compatible distractors, action observation is thought to facilitate motor or production mechanisms required for performing the prompted action, thus reducing response times (RTs) and errors. In contrast, incompatible distractors result in competition between the facilitated motor mechanisms and those required to produce the prompted response, thus increasing RTs and errors. A larger automatic imitation effect (i.e., a larger RT difference between incompatible and compatible trials) indicates that production mechanisms were more engaged for the distractor, thus showing a measure of covert imitation (Heyes, 2011). In vocal SRC paradigms, participants produce a speech response following a prompt (e.g., say “ba” when seeing “£”) while ignoring a distractor (e.g., an audio recording and/or a video of someone saying “ba”). As for manual SRC tasks, RTs are slower for incompatible (“da”) than compatible (“ba”) distractors (Galantucci et al., 2009; Kerzel & Bekkering, 2000; Roon & Gafos, 2015).

The integrated theory of language production and comprehension proposes that covert imitation serves a specific purpose in action processing (Pickering & Garrod, 2013). When planning to produce a speech action (e.g., an instruction to say “ba” or “da”), a set of motor (articulatory) commands is formulated plus two control signals. A perceptual control signal processes the proprioceptive (and/or visual/auditory) experience in the speech production system of the action being executed. This perceptual signal is used as sensory, or reafferent, action feedback. The second control signal is an efference copy of the action ultimately resulting in a predicted percept of producing the planned utterance. The perceptual feedback signal and the predicted percept are compared in real time during action execution. Whenever a discrepancy is detected, an error signal is sent to the action planning mechanism to update motor plans.

When perceiving speech, a similar mechanism is presumed to operate, except, here, forward models generate predictions regarding upcoming speech utterances. Pickering and Garrod (2013) propose that this predictive process operates via the simulation or the association route. The simulation route is based on listeners’ experiences of producing speech utterances. Here, listeners use a process of covert imitation to generate a forward perceptual model. This covert imitation crucially involves the engagement of forward models predicting incoming speech and the engagement of production mechanisms. The association route is based on listeners’ experience of perceiving others’ utterances. This route also involves engagement of prediction via forward models, except the links to sensory processing systems are activated rather than production systems. The simulation route is preferred when the speaker is similar/familiar (e.g., culturally, speech production style, anatomy) to the listener. When the speaker is less similar (e.g., when they are a nonnative speaker), the association route is used.

The integrated theory predicts that observing speech utterances produced by a similar or familiar a speaker involves covert imitation, as it will preferentially engage the simulation route (Adank et al., 2010). Listening to a speaker who is dissimilar or unfamiliar will engage less covert imitation, as it will preferentially engage the association route. The integrated theory predicts that speech utterances for dissimilar speakers will vary in evoked covert imitation. Listening to similar speakers will evoke larger automatic imitation effects than listening to less similar speakers. Thus far, no speech SRC studies have been conducted to test this prediction, but various SRC studies using manual actions tested a related question—namely, whether observation of computer-generated manual actions results in less automatic imitation compared with human-produced actions (Cracco et al., 2018; Gowen & Poliakoff, 2012; Longo et al., 2008; Press et al., 2005; Press et al., 2006; Stürmer et al., 2000).

Press et al. (2005) presented participants with an opening or closing hand produced by a human or a robot. They found more automatic imitation for human stimuli (33-ms automatic imitation effect) versus robotic stimuli (6 ms). When testing was repeated on the second day, automatic imitation decreased for human stimuli (24 ms) and increased for robotic stimuli (11 ms). Press et al. concluded that decreased automatic imitation for robotic stimuli shows that human movement stimuli are more effective visuomotor primes than robotic stimuli. Press et al. (2006) manipulated stimuli depicting a human hand by adding a metal wire wrist and informed participants that these stimuli were produced by a robot. Participants in Experiment 1 produced a prespecified response (hand opening/closing) for compatible and incompatible distractors while RTs were measured as in Press et al. (2005). They report no differences between SRC effects for human (16 ms) and robotic (26 ms) stimuli. Experiment 2 aimed to disentangle beliefs about the stimuli from stimulus animacy. Participants in a between-group design saw a genuine human or robotic hand (blue animated silhouettes) producing the actions. Participants presented with the genuinely human stimulus were told that the hand was either human or robotic in the two sessions of testing, and participants who were presented the genuinely robotic stimulus were told that the movement was generated by human or robotic movement. The participants who saw genuine human stimuli displayed similar automatic imitation for stimuli they were told were human (15 ms) and for stimuli they were told were robotic (14 ms). Participants who saw genuine robotic stimuli showed similar automatic imitation for stimuli they thought were human (5 ms) and for stimuli they believed to be robotic (5 ms). Experiment 2 demonstrated that stimulus properties modulate automatic imitation, with less automatic imitation for genuinely robotic stimuli.

Longo et al. (2008) presented participants with two sets of computer-generated, human-simulating manual stimuli: biomechanically possible and impossible hand actions. The possible actions consisted of a hand lowering either the index or middle finger. The impossible actions consisted of a hand with the index of middle finger bending in an impossible angle. In Experiment 1, participants completed an SRC task that included both types of stimuli, without any instructions regarding the difference between possible and impossible actions. They found similar automatic imitation for possible (7 ms) and impossible (8 ms) actions. In Experiment 2, participants were informed that some finger movements were possible, and some were impossible. They found that automatic imitation for impossible stimuli nearly disappeared (1 ms), while automatic imitation persisted for possible movements (10 ms). Longo et al. show an effect of beliefs on automatic imitation of impossible actions not reported by Press et al. (2006).

Nevertheless, while it has been established that automatic imitation is generally decreased for nonhuman- than for human-generated manual actions, it is not evident that vocal actions show the same pattern in automatic imitation of actions with a different biological origin as manual actions. The current study aimed to address this gap and test if computer-generated vocal stimuli evoke decreased automatic imitation compared with human-produced vocal stimuli. Computer-generated, or synthetic, utterances are ideal for testing the prediction of the integrated theory regarding the use of covert imitation during perception. Computer-generated speech, especially when generated by a less sophisticated speech synthesizer, represents a speaker who will be dissimilar to the listener. Synthetic speech is generated by a speech synthesis programme running on a computer, which is physically and conceptually dissimilar from a human speaker. Consequently, the integrated theory predicts more covert imitation when listening to a human speaker for computer-generated speech. We conducted an online SRC experiment in which participants were presented with syllables (“ba” and “da”) produced by a male human speaker or generated by a computer programme. Based on the integrated theory and results from Press et al. (2005; Press et al., 2006), we predicted that automatic imitation would vary depending on the speaker’s biological status and be decreased for computer-generated speech.

## Method

### Participants

The study was conducted online, participants were recruited on Prolific (prolific.co) and the experiment hosted on Gorilla (gorilla.sc). We used the Bayes stopping rule to determine our sample size. We set our minimum sample size to 32 participants; the number required to fully counterbalance the design. After this minimum sample was collected, we calculated the BF10 based on model fit (Jarosz & Wiley, 2014) for a model including the two-way interaction between compatibility and speech type versus one including only the main effects. BF10 > 3 were considered evidence in favour of the alternative hypothesis, and we considered BF10 < 0.2 as evidence in favour of the null hypothesis (Raftery, 1995). Participants had completed a minimum of five studies on Prolific with an approval rate of ≥95% and were required to run the study on a computer, using Chrome, with wired headphones. A total of 191 participants were recruited for the eligibility screening, to obtain the final sample of 32 participants (16 female, average age = 23.7 years, *SD* = 3.6 years, range: 18–31 years). Exclusions are listed in detail in the Supplementary Materials. Participants received £0.75 upon completion of the eligibility study, and those who passed the eligibility study received a further £4 upon completion of the main task, a rate commensurate to £7.50 per hour. The experiment was approved by the Research Ethics Committee of University College London (UCL) and conducted under Project ID #15365.001.

### Materials

The human SRC stimuli were audio recordings of a 40-year-old male British-English speaker from the North-West of England saying /ba/ and /da/ in a neutral tone of voice. The recording were made using a RØDE NO1-A Condenser Microphone and a Focusrite Scarlett 2i4 USB Computer Audio Interface preamplifier plugged into the sound card input of a Dell PC in a sound-proofed room at 44.1 kHz. Audio recordings were amplitude-normalized, down-sampled to 22.050 kHz, saved as mono, and scaled to 70 dB SPL (sound pressure level) using Praat (Boersma & Weenink, 2018).

The computer-generated vocal stimuli were created using a Klatt synthesizer (Klatt, 1980) using in-house software. We decided against the use of a modern, sophisticated speech synthesizer, which can produce speech near-indistinguishable from human-produced speech utterances (Wagner et al., 2019). In contrast, the Klatt synthesizer and other synthesizers developed in the 1980s (e.g., MITtalk, DECTalk; successors of the Klatt synthesizer) produce speech that is clearly distinguishable from human-produced speech (Pisoni et al., 1985; Ralston et al., 1991).

Using the Klatt synthesizer, we constructed a continuum between /ba/ and /da/ in 10 steps. All computer-generated syllables were converted to .wav files and matched to the human speech sounds as much as possible (Table 1) in terms of their sampling frequency, intensity, average fundamental frequency (*f*_*o*_*)*, and lowest three formant frequencies. We matched the acoustics characteristics of the human and computer-generated stimuli closely to avoid possible confounds introduced by a difference in intelligibility between the two stimulus types, or by paralinguistic issues such as perceived accent. We ran a pilot study (*N* = 10, not reported) to establish which of the computer-generated syllables were most frequently classified as “ba” and “da.” The two selected stimuli were classified as /ba/ and /da/ in >95% of instances.

**Table 1:** Stimulus characteristics of human and computer-generated stimuli

	Human /ba/	Human /da/	Computer-generated /ba/	Computer-generated /da/
Duration	473 ms	473 ms	470 ms	470 ms
Intensity	75 dB SPL	75 dB SPL	75 dB SPL	75 dB SPL
Average f_o_	121.9 Hz	123.2 Hz	131.8 Hz	131.6 Hz
Average F_1_	661.8 Hz	656.6 Hz	761.6 Hz	768.5 Hz
Average F_2_	1040.3 Hz	1042.3 Hz	1150.9 Hz	1178.1 Hz
Average F_3_	2553.6 Hz	2595.5 Hz	2458.2 Hz	2559.1 Hz
Consonant duration	37 ms	42 ms	34 ms	39 ms
Vowel duration	435 ms	420 ms	436 ms	431 ms

F = formant frequency; *f*_o_ = fundamental frequency; dB = decibel; SPL = sound pressure level

**Fig. 1:**
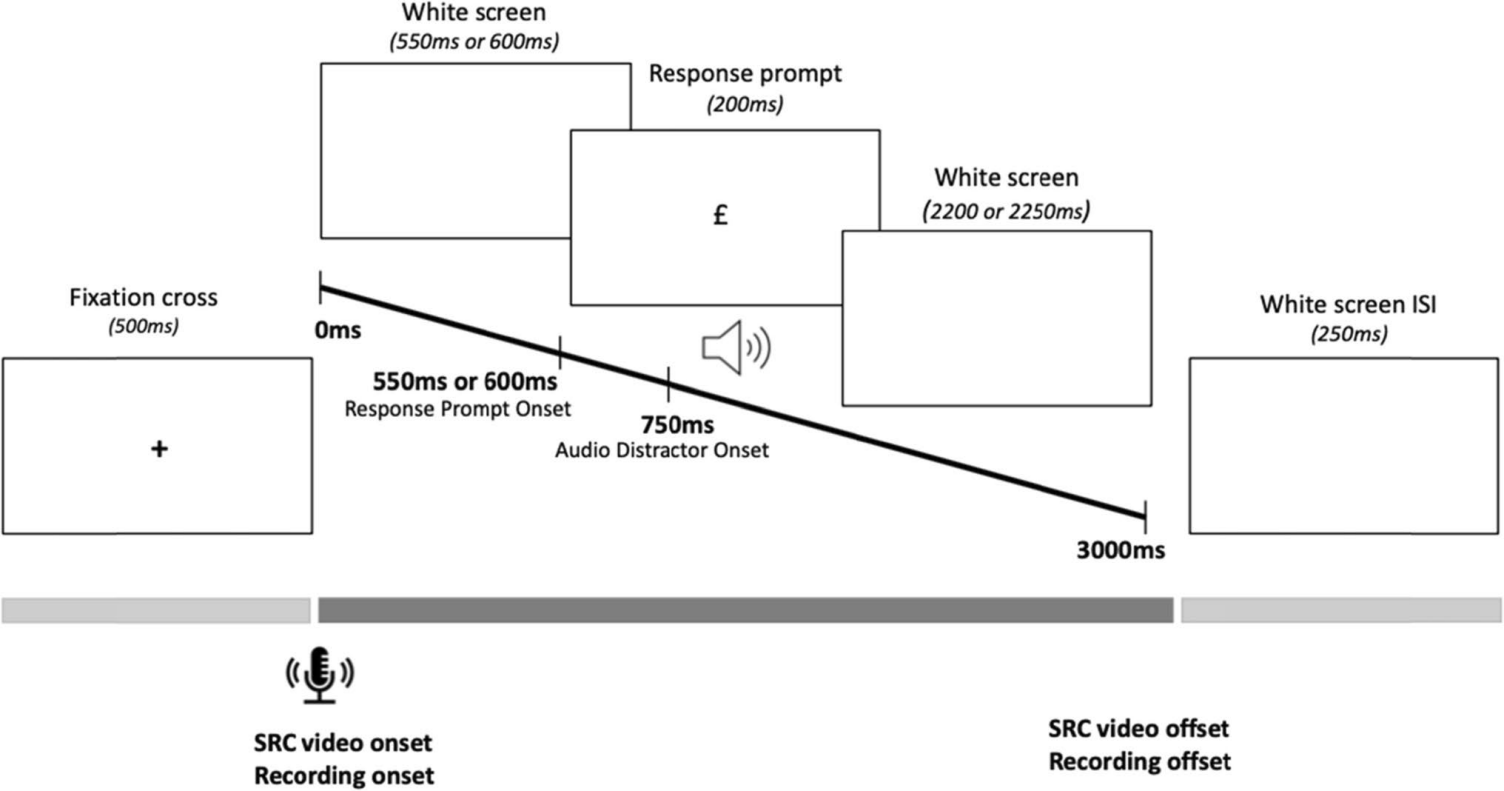
Trial design for the SRC task. On Gorilla, a 500-ms fixation cross was created to precede a 3,000-ms SRC video, followed by a 250-ms interstimulus interval (ISI). Audio recordings were set to co-occur with SRC video presentation. Timings in parentheses indicate durations of specific events. The response prompt appeared at either 550 ms (SOA1) or 600 ms (SOA2) from SRC video onset

Materials consisted of audio clips of speech sounds /ba/ and /da/, and response prompts “&” and “£” (Arial, font size 48). Stimulus videos were generated in Microsoft PowerPoint and included both the prompt and the distractor audio signal to align audio stimuli and visual prompts precisely, to overcome stimuli onset lags observed in online experiments (Bridges et al., 2020). We presented the prompt at two stimulus-onset asynchronies (SOAs) relative to auditory distractor onsets. The SRC videos started with a blank screen, followed by the presentation of the symbol prompt (& or £) in the centre of the screen at 550 ms or 600 ms for 200 ms. The audio syllable started playing 750 ms from the start of the video, for 473 ms for the human stimuli and for 470 ms for the computer-generated stimuli. The video stopped playing after 3,000 ms had elapsed. We chose to use negative SOAs of −200 (SOA1) and −150 ms (SOA2) relative to the presentation of the audio distractor (Ghaffarvand Mokari et al., 2020, 2021; Roon & Gafos, 2015). This procedure ensured that the distractor stimuli would be presented at a time point at which participants would most likely be preparing their response. Thus, SOA1 started at 550 ms relative to video onset, and 200 ms before the audio start of the audio distracter, and SOA2 was presented 600 ms relative to video onset and 150 ms before the audio distracter. Note that we use ‘*SOA’* to indicate the onset of the prompt relative to distractor onset, following Adank et al. (2018), Kerzel and Bekkering (2000) and Wu et al. (2019). Other SRC studies have reversely defined SOA as the onset of the distractor relative to response prompt onset (Galantucci et al., 2009; Ghaffarvand Mokari et al., 2020, 2021; Roon & Gafos, 2015). Negative SOAs in the current experiment hence indicated that response prompts appeared before the auditory distractor. On Gorilla, the video was preceded by a 500-ms fixation cross and followed by a 100-ms interstimulus interval (ISI). Recordings were set to start with distractor video onset and last 3,000 ms. The trial design for the SRC task is detailed in Fig. 1.

To ensure that participants were paying attention and did not mute their sound, we included a “catch” trial in each block of the SRC task. In each catch trial, participants heard tone stimuli consisting of one, two, or three tones and were asked to report how many tones they had heard via button press. Using Praat (Boersma & Weenink, 2018), we created a 350-Hz tone with a length of 200 ms, with a sampling frequency of 44.1k Hz, saved as a mono sound. We created three .wav files for the catch trials: The first consisted of a single tone, the second of two tones spaced 32-ms apart, while the third file contained three tones, all spaced 32-ms apart.

### Procedure—Eligibility screening

Participants first completed an eligibility screening study to assess the adequacy of their hardware in providing quality audio recordings. In this eligibility study, participants read the information sheet and provided consent before completing a headphone check (Woods et al., 2017). In each of six trials, participants judged which of three pure tones was the quietest. Performance below 83% correct (chance level: 33.33%) led to the immediate rejection of participants from the experiment. Participants having passed the headphone check then completed an audio recording task. In all eight trials, participants viewed a fixation cross for 500 ms followed by an SRC video without the symbol prompt. 1,500 ms from trial onset, participants produced either /ba/ and /da/ as indicated by written instructions on the screen, as quickly as possible. For 16 further trials, participants were instructed to remove their headphones and placed them close to their microphone and turning their system volume to maximum. Trials began with a 500-ms fixation cross, followed by an SRC video (3,000 ms) and a 100-ms interstimulus interval (ISI). Recordings were set to start at video onset and last 3,000 ms. This task was included to collect recordings of the participants’ voice to check sound quality. Participants were considered eligible if they followed task instructions and provided good-quality audio recordings, if audio stimuli onsets recorded from their headphones in the final 16 trials fell within an 80-ms range, and if recordings were consistently clear of static or excessive background noise.

### Procedure—Main task

Participants who passed the eligibility test were invited to take part in the main experiment. After reading the information sheet and providing consent, participants completed the headphone check as per the eligibility study. Participants then completed a two-alternative forced-choice (2AFC) phonetic identification task where they classified all four auditory stimuli (computer-generated /ba/ and /da/ and human /ba/ and /da/) as /ba/ or /da/. This task was included to establish the relative intelligibility of both types of stimuli. Each trial in the 2AFC task started with a 250-ms fixation cross, followed by a 100-ms ISI after which an audio file played. Participants indicated via key press (left or right arrow key) which sound they heard. Human and computer-generated stimuli were presented in separate 2AFC blocks. Each block consisted of two practice trials and 20 main trials (2 syllables × 10 repetitions). After the 2AFC task, participants moved on to the SRC task. They first read detailed instructions on the task, including prompt–response pairings and instructions to produce the prompted response as quickly and accurately as possible. Participants were informed that they would hear distractor sounds that were produced by a male human speaker or computer generated and were provided with examples of both types of stimuli. Participants were also informed of the catch trials. They then completed an SRC block with human stimuli and an SRC block with computer-generated stimuli, in randomized order. Before either task, participants were reminded of the prompt-response pairings and completed eight practice trials (2 prompts × 2 distractors × 2 SOAs). The main SRC task consisted of five blocks of 24 trials. The mapping of the prompt (& or £) onto the response (ba or da) was counterbalanced across participants, who were reminded of the prompt–response mappings between blocks.

A catch trial was included within each block. Catch trials started with a 500-ms fixation cross, after which one of the catch trial auditory stimuli was played and participants indicated via button press whether they heard one, two, or three tones. A minimum of 75% correct catch trials was required to be included in the final data analysis.

After the SRC task, participants completed a video onset detection test aimed at estimating latencies between recording onsets and SRC video distractor onsets. Participants received the same instructions as in the second part of the eligibility test (i.e., to place their headphones close to their microphone and turn their system volume to maximum so the sound of the stimuli could be recorded from their headphones). As in the SRC tasks, audio recordings were set to start with SRC video onset and last 3,000 ms. Here, each SRC trial type (2 conditions × 2 prompts × 2 syllables × 2 SOAs) was presented four times and recorded, for a total of 64 trials. The average delay between the expected audio onset in the SRC video stimuli (750 ms) and the recorded audio onset obtained in the recordings of the video onset detection task was used to adjust reaction time (RT) estimations for the SRC tasks.

### Data processing and analysis

Responses were recorded via the audio recording zone on Gorilla.sc. Recordings were initially encoded as stereo .weba files and recoded offline as mono .wav files. Responses were coded manually and RTs annotated manually on Praat (Boersma & Weenink, 2018). The syllable detection script from the Prosogram plugin (Mertens, 2004) was first used to delimitate participants’ productions for each recording (i.e., for each trial). The acquired boundaries were checked and adjusted manually to account for noise and detection errors. While audio recordings were set to coincide with the presentation of SRC video stimuli, piloting revealed delays between recording and video onsets. This asynchrony was problematic, as latencies in video stimuli onsets—and hence response prompt onsets—inflated RT estimations. To estimate these video onset latencies and correct RT computations, audio onsets were measured for each trial in the video onset detection test and compared with the audio onset in the video stimuli (750 ms). Mean video onset latencies (*measured audio onset − expected audio onset [750 ms]*) were computed for each participant and combination of syllable (ba or da), biological status, and SOA. Video onset latencies averaged 118 ms across participants (*SD* = 49 ms, range: 43–295 ms). We also computed standard deviations of the video onset latencies for each combination of participant, syllable, biological status, and SOA as an indicator of the variability in video onset latencies per condition. These standard deviations averaged 12 ms. To obtain RTs from prompt onsets in the SRC task, RTs measured manually from recording onsets were corrected for SOA (550 ms or 600 ms from video onset) as well as for mean video onset latencies (*RT from prompt onset* = *measured RT from recording onset* – *SOA* – *mean video onset latency*).

Data were analyzed with generalized linear mixed-effects models (GLMMs) using the *lme4* package in R (Bates et al., 2014). GLMMs enable the modelling of independent variables of interest (*fixed* effects) whilst considering unexplained variability within and across participants (*random* effects). A further appeal of GLMMs is that they allow for the analysis of non-normally distributed data through specifications of a distribution and link function, which is considered preferable to transformation for RT data (Balota et al., 2013; Lo & Andrews, 2015; Schramm & Rouder, 2019). In addition, a further advantage was that this analysis allowed us to avoid potential issues reported with log-transforming and subsequently back transforming RT data (Feng et al., 2013; Lo & Andrews, 2015; Manandhar & Nandram, 2021; Molina & Martín, 2018). Instead, we chose to use a gamma-distribution and identity link function to account for positive skew in RT data without the need to transform the data, following Lo and Andrews (2015).

In the current experiment, fixed effects were compatibility (compatible vs. incompatible), biological status of the distractor stimulus (human vs. computer generated), SOA (SOA1 vs. SOA2), and their interactions. The random effect structure consisted of by-participant intercepts. Errors were excluded from the RT analyses. Errors included productions of wrong or multiple responses, missing answers, anticipatory responses (RT <200 ms) and late responses (RT >1,000 ms). For each participant, observations with RTs outside of three median absolute deviations (MADs) from their median RT in each condition (combination of biological status, compatibility, and SOA) were removed from the analyses.

The error analyses used a binomial distribution and logit link function. We used a forward model building strategy to determine the best fitting model. Starting with a model with random effects only, we performed chi-squared tests to assess improvement of model fit after the inclusion of each fixed factor, from lower order to higher order effects. A factor was only maintained in the model if it significantly improved model fit. To supplement our analyses, Bayes factors (BF_10_) were computed at each step following Jarosz and Wiley (2014) to evaluate evidence for each factor. BF_10_ quantifies the likelihood of the alternative hypothesis (H_1_) over the null hypothesis (H_0_). Further, Cohen’s *d* (Cohen, 2013) was computed to estimate the effect size of the significant effects in the final model using the following formulas (Equations 1 and 2).

1 $$Cohe{n}^{\prime }s\ d=\frac{\left({M}_2-{M}_1\right)}{Pooled\ SD}.$$

2 $$Pooled\ SD=\frac{\sqrt{{SD_1}^2+{SD_2}^2}}{2}.$$

## Results

The full data set for 32 participants consisted of 7,667 observations. For the reaction time (RT) analyses, erroneous trials were removed (687 trials, 8.96%). These included 252 erroneous responses, five missing answers, six anticipatory responses, and 424 late responses. We removed 567 observations with RTs outside of three median absolute deviations (MADs) from each participant’s median at each experimental level (i.e., each combination of biological status, compatibility, and SOA). The remaining 6,413 trials were included in the RT analyses. The final model included the main effects compatibility, biological status, and SOA, as well as the interaction between compatibility and SOA (Table 2; see the Supplementary Materials for the model selection process).

**Table 2:** Mean raw reaction times (RTs) and standard deviations (*SD*s) in milliseconds (ms) per condition

Biological Status	SOA	Compatibility	*M* (ms)	*SD* (ms)
Human	SOA1	Compatible	686	143
		Incompatible	699	141
	SOA2	Compatible	672	128
		Incompatible	693	131
Computer-generated	SOA1	Compatible	684	139
		Incompatible	686	138
	SOA2	Compatible	662	131
		Incompatible	679	121

*SOA* stimulus-onset asynchrony

**Fig. 2:**
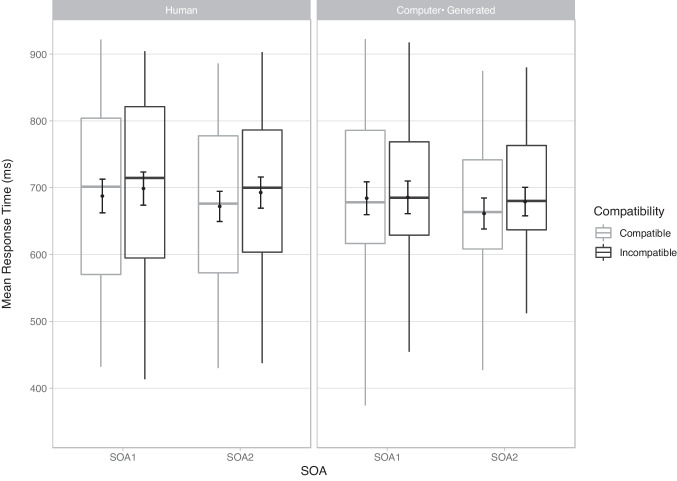
Mean response times (RTs) in milliseconds (ms) for correct trials in the stimulus–response compatibility (SRC) tasks for each experimental condition. Points in the background show the raw mean RTs for each participant (points are offset on the *x*-axis for clarity). The boxplots indicate the first, second (median), and third quartiles and whiskers indicate 1.5 times the interquartile range of the distribution. Black points in the foreground show the mean and error bars indicate standard errors

There was a significant main effect of compatibility with slower RTs for incompatible trials (*M* = 689 ms, S*D* = 132 ms) than for compatible trials (*M* = 676 ms, *SD* = 134 ms), BF_10_ = 3269017, *d* = 0.10. The results showed an overall automatic imitation effect of 13 ms (Fig. 2). The main effect of biological status was also significant, with slower RTs in response to human (*M* = 688ms, *SD* = 135ms) than to computer-generated stimuli (*M* = 678 ms, *SD* = 131 ms), BF_10_ = 665.14, *d* = 0.08. The main effect of SOA was significant, with slower RTs at SOA1 (*M* = 684 ms, *SD* = 154 ms) than at SOA2 (*M* = 674, *SD* = 144 ms), BF_10_ = 54.60, *d* = 0.07. The effect of compatibility was modulated by SOA, with larger automatic imitation effects at SOA2 (*M* = 19 ms, *SD* = 27 ms) than at SOA1 (*M* = 6 ms, *SD* = 27 ms), BF_10_ = 20.09, *d* = 0.48. Crucially, there was no evidence for an interaction between compatibility and biological status with similar automatic imitation effects (incompatible minus compatible RTs) in the human (*M* = 16 ms, *SD* = 28 ms) and in the computer-generated (*M* = 10 ms, *SD* = 27 ms) condition, BF_10_ = 0.02. The low BF_10_ provides strong evidence for the null hypothesis. Results for the errors and the 2AFC task can be found in the supplementary materials, and while there was a significant difference in identification of human and computer-generated speech, both were extremely high and well above chance (>90%).

## Discussion

The present study aimed to establish whether computer-generated vocal actions evoke an automatic imitation response. Second, it aimed to determine whether this evoked response was smaller or similar to responses measured for human vocal actions. Participants responded slower and with more errors to incompatible trails than to compatible trials, displaying a clear automatic imitation effect of 13 ms. Participants also responded slower (and with lower accuracy) to the human-produced vocal actions than the computer-generated actions. There were no further significant main effects or interactions, showing that human vocal stimuli are not treated differently in terms of automatic imitation of vocal actions by the speech perception system as proposed by the integrated theory. There was a trend towards a smaller automatic imitation effect for the computer-generated vocal stimuli, but adding the interaction between compatibility and biological status did not improve model fit.

In contrast to Longo et al. (2008), we do not find any effects of top-down manipulation. Press et al. (2005, 2006) report no effect of informing participants about the human or robotic status of their stimulus materials and neither did we find a difference in automatic imitation effects between the two stimulus types, despite informing participants that the stimuli were produced by a human or computer generated. Based on Press et al. (2005, 2006) and Longo et al. and predictions from the integrated theory, we expected smaller automatic imitation effects for the computer-generated vocal stimuli, but our results did not confirm this prediction.

### Integrated theory of language production and comprehension

Results did not confirm the prediction from the integrated theory that covert imitation is reduced for speakers less similar to the listener. One possibility is that both types of stimuli were perceived as equally similar to the listener and therefore both processed via the simulation pathway, as the result of a learning effect. Participants may have become overly familiarized during the 2AFC identification task (including twenty repetitions of the computer-generated stimuli) before the main SRC task. We therefore conducted an additional analysis (Supplementary Materials) to evaluate whether participants showed changes in the size of the automatic imitation effect over the course of the SRC task, but we found this effect remained stable. Therefore, it seems unlikely that any learning effects—participants getting more accustomed to the computer-generated stimuli—affected results. In addition, it seems likely the computer-generated stimuli were perceived as dissimilar to the human actions, as they were classified correctly less often than the human actions, as also reported in (Pisoni et al., 1985) for speech stimuli generated using comparable speech synthesizers.

An alternative explanation is that the theory’s predictions are incorrect (or underspecified with respect to similar, dissimilar, familiar, or less familiar speech). For instance, it could be the case that all vocal stimuli are processed using the simulation route only. Using a single pathway for all incoming speech would be more parsimonious compared with two pathways, as no mechanism is needed to decide whether an incoming vocal stimulus is similar enough to be processed through the simulation route or whether needs to be processed using the association route. Processing of dissimilar speakers might thus rely as much on engagement of production mechanisms as familiar speakers, in contrast to predictions of the integrated theory.

### Automatic imitation of vocal versus manual actions

Our results did not replicate Press et al. (2005, 2006), who report smaller automatic imitation effects for computer-generated manual stimuli, whereas we did not. This discrepancy could be due to differences in how computer-generated manual and vocal stimuli are perceived. Manual computerized actions (e.g., the pincher used in Press et al., 2005, 2006) may be processed as inanimate objects such as tools, while vocal computer-generated actions may instigate us to assign an identity to its bearer, as for faces (Lavan et al., 2019). While it is feasible to manipulate manual stimuli to evoke specific stereotypes (e.g., by using racial cues; cf. Correll et al., 2015), we did not explicitly implement such a manipulation and neither did we ask participants whether they assigned an identify to either type of stimulus. It is also unclear whether hands evoke stereotypical connotations as is the case for voices. Upon hearing an unfamiliar voice, listeners create a mental image of the speaker’s physical appearance (Krauss et al., 2002), and imagine the physical appearance of computer-generated voices (McGinn & Torre, 2019). Moreover, voice identity perception studies demonstrated that listeners attribute traits (trustworthiness or aggression) to unfamiliar voices after brief exposures (100–400 ms; Mileva & Lavan, 2022). It is unclear whether listeners also attribute voice identity traits to computerized voices. Therefore, it seems plausible that the difference between our results and those reported in Press et al. (2005, 2006) is due to differences in how manual and vocal stimuli evoke stereotypical notions related to their origin or bearer.

### Limitations and future directions

The Klatt synthesizer used was unsophisticated compared with current speech synthesizers (Wagner et al., 2019). We intended to establish a baseline of the capacity of synthetic stimuli to evoke an automatic imitation effect, so we selected a synthesizer that created synthetic-sounding stimuli, so stimuli were not confused with human-produced stimuli and avoid a situation where participants believed that both stimuli were human produced. We intended to keep stimulus intelligibility stable for human and computer-generated conditions. The computer-generated stimuli were less intelligible than human stimuli (96.48% computer-generated stimuli vs. 99.53% human stimuli; cf. Supplementary Materials). Future experiments could first disentangle biological status and intelligibility, as these factors were confounded in our study. For instance, human and computer-generated stimuli could be degraded parametrically using noise. Using computer-generated and human stimuli equalized for intelligibility, it would be feasible to also introduce a top-down manipulation (e.g., by informing one group of participants that all stimuli are human vs. telling a different group that all stimuli are computer-generated). This way, separate effects of top-down (stimulus beliefs) and bottom-up (intelligibility) on automatic imitation of vocal actions could be established. Second, future experiments could clarify the relationship between familiarity/similarity and biological status. For instance, per the integrated theory, processing of computer-generated speech matching the listener’s regional or foreign accent could rely more on the simulation than on the association route and vice versa. An experiment with a two-factor factorial design with familiarity and biological status as factors could establish whether this prediction can be confirmed.

Participants responded 10ms faster to computer-generated trials. This effect counters findings for similar condition-related effects reported in Press et al. (2006) and Longo et al. (2008), who found slightly faster RTs (Press et al., 2006) for human compared with computer-generated stimuli or no RT difference between impossible and possible actions (Longo et al., 2008). We suspect that this difference between our study and the studies might modality specific. Perhaps observers respond differently to manual and speech stimuli differing in biological status, because our speech stimuli can be categorially perceived (Liberman et al., 1967) and the manual stimuli used in Press et al. (2006) and Longo et al. cannot. This possibility could be explored in future experiments—for example, using a factorial design with modality (manual or speech responses) and biological status (human and computer-generated) fully crossed.

## Conclusions

Our results demonstrated that vocal stimuli from human and non-human origins evoke similar covert imitation. The speech perception system is therefore not tailored towards vocal stimuli produced by fellow humans and may treat any speech-like signal the same. Our results further demonstrate that computer-generated vocal stimuli evoked a covert imitative response. Finally, our results have implications for the integrated theory of language comprehension, but also for research in human-computer interactions and voice identity research.

## Supplementary information

ESM 1 (DOCX 25 kb)
