# Supplementary material for: Automatic imitation of human and computer-generated vocal stimuli
Source: Psychon Bull Rev. 2022 Nov 28;30(3):1093–102. doi: 10.3758/s13423-022-02218-6 (PMC10264473; doi:10.3758/s13423-022-02218-6)
Supplement: Supplementary file 1 — (DOCX 25 kb) [file 13423_2022_2218_MOESM1_ESM.docx]

**Appendix A**. Exclusion number for experimental testing stage.

**Table A1.** Exclusion numbers and criteria at each stage of the experiment.

| Experiment stage | Starting *n* | Exclusion criteria | *n* exclusions |
| --- | --- | --- | --- |
| Eligibility screening | 191 | Failed headphone check  Data collection error  Beyond age limit  Missing data  Low quality audio  Did not follow instructions  **Total exclusions** | 45  2  9  21  37  2  **116** (60.73%) |
| Main study | 75 | Did not start the main experiment  Failed the headphone check  Did not finish the experiment  **Total exclusions** | 30  2  3  **35** (46.67%) |
| Data processing | 40 | Missing data  Accuracy <50% in the SRC task  Error rate >3 SD from group mean  Accuracy <70% for >=1 sound in the 2AFC task  Failed the catch trials  **Total exclusions** | 1  3  0  4    0  **8** (20%) |
| *Note.* *n* = participant number. Final *n* = 32. | | | |

**Appendix B.** Forward model selection procedure for the reaction time (RT) analyses.

*Reaction Time (RT) analyses*

We first assessed the effect of compatibility by comparing it with a model including only the random effects of participant. Inclusion of Compatibility significantly improved model fit (χ^2^(1)=39.061, *p*=4.107 x 10^-10^, BF_10_=3269017), reflecting the longer RTs for incompatible (*M*=689ms, S*D*=132ms) than compatible (*M*=676ms, *SD*=134ms) trials. Including the main effect of SOA increased model fit (χ^2^(1)=16.994, *p*=3.75 x 10^-5^, BF_10_=54.598), with faster RTs at SOA2 (*M*=674, *SD*=144ms) than SOA1 (*M*=684ms, *SD*=154ms). The main effect of Biological Status improved model fit (χ^2^(1)=21.448, *p*=3.636 x 10^-6^, BF_10_=665.142), reflecting with slower RTs in response to human (*M*=688ms, *SD*=135ms) than to computer-generated stimuli (*M*=678ms, *SD*=131ms). The two-way interaction between compatibility and SOA further improved model fit (χ^2^(1)=15.036, *p*=0.0001, BF_10_=20.086), with larger compatibility effects at SOA2 (*M*=19ms, *SD*=27ms) than at SOA1 (*M*=6ms, *SD*=27ms). Crucially, adding the interaction between Compatibility and Biological Status did not improve model fit (χ^2^(1)=0.425, *p*=0.515, BF_10_ =0.018). The BF_10_ value indicated that the alternative hypothesis – that Biological Status affects Compatibility effect – was 0.018 times as likely as the null hypothesis, i.e., the null hypothesis was 54.60 times more likely (1/BF_10_). Adding the two-way interaction between Biological Status and SOA did not improve model fit (χ^2^(1)=0.425, *p*=0.515, BF_10_=0.018), neither did including the three-way interaction between Compatibility, Biological Status and SOA (χ^2^(5)=2.887, *p*=0.409, BF_10_=1.013 x 10^-5^). The final model (Table B1) therefore included random by-participant intercepts and main effects of Compatibility, SOA, Biological Status and the two-way interaction between Compatibility and SOA.

**Table B1.** Final model of raw reaction times (RTs) in milliseconds (ms) using a gamma distribution and identity link function.

| Fixed Effect | Estimate | *SE* | t-value | p-value | BF_10_ |
| --- | --- | --- | --- | --- | --- |
| **(Intercept)** | **718.063** | **6.222** | **115.404** | **< 2 x 10^-16^***** |  |
| **Compatibility** | **5.120** | **2.457** | **2.084** | **0.037*** | **3269017** |
| **Biological Status** | **-16.661** | **2.404** | **-6.929** | **4.24 x 10^-12^***** | **665.142** |
| **SOA** | **-9.931** | **2.042** | **-4.863** | **1.16 x 10^-6^***** | **54.598** |
| **Compatibility x SOA** | **16.435** | **3.392** | **4.845** | **1.27 x 10^-6^***** | **20.086** |
| *Note:* SOA = Stimulus-Onset Asynchrony. Baseline conditions: compatible, biological status, SOA1. * *p*<.05, ***p*<.01, ***p<.001. | | | | | |

**Appendix C.** Error and 2AFC analyses.

*Error analyses*

All 7667 observations were used in the accuracy analyses. Results of the chi-squared tests at each step of the model building process are presented in Appendix B. The final model included the main effects of Compatibility and Biological Status, cf. Table 5. Both effects were significant, with more errors for incongruent (*M*=10.15%, *SD*=1.36%) than for congruent (*M*=7.79%, *SD*=1.07%) trials, and in the human condition (*M*=9.83%, *SD*=1.59%) compared to the computer-generated condition (*M*=8.10%, *SD*=1.49%). Note that the low BF_10_ for the effect of Biological Status (BF_10_= 0.522) suggests that evidence for the effect is weak. Once again, there was no evidence for an interaction between congruency and condition, with strong evidence for the null hypothesis (BF_10_= 0.013). See Appendix B for full details of the analysis of the errors.

**Figure B1**. Mean error rates (ERs, %) in the stimulus-response compatibility (SRC) tasks for each experimental condition. Points in the background show the raw mean ERs for each participant (points are offset on the x-axis for clarity). The boxplots indicate the first, second (median) and third quartiles, and , and whiskers indicate 1.5 times the interquartile range of the distribution. Black points in the foreground show the mean and error bars indicate standard errors.

*Model selection for error analyses.*

Starting with a model with only random effects of participant, including the effect of Compatibility significantly improved model fit (χ^2^(1)=13.89, *p*=0.0002, BF_10_=11.588), reflecting the higher error rates (ERs) for incompatible (*M*=10.15%, *SD*=1.36%) than compatible (*M*=7.79%, *SE*=1.07%) trials. Including the main effect of SOA did not improve model fit (χ^2^(1)=0.294, *p=* 0.588, BF_10_=0.014). The main effect of Biological Status improved model fit (χ^2^(1)=7.617, *p*=0.006, BF_10_=0.522), reflecting larger ERs when responding to human (*M*=9.83%, *SD*=1.59%) than to computer-generated stimuli (*M*=8.10%, *SE*=1.49%). Note that the low BF_10_ fo suggests that evidence for the effect of Biological Status is weak. The two-way interaction between compatibility and SOA model did not enhance model fit (χ^2^(1)=0.509, *p*=0.775, BF_10_=0.0002). Crucially, adding the interaction between Compatibility and Biological Status did not improve model fit (χ^2^(1)=0.272, *p*=0.602, BF_10_ = 0.018). The BF_10_ value indicated that the alternative hypothesis – that Biological Status affects the Compatibility effect – was 0.018 times as likely as the null hypothesis, i.e., the null hypothesis was 55.56 times more likely (1/BF_10_). Adding the two-way interaction between Biological Status and SOA did not improve model fit (χ^2^(1)=2.067, *p*=0.356, BF_10_=0.0004), neither did including the three-way interaction between Compatibility, Biological Status and SOA (χ^2^(5) = 2.887, *p* = 0.409, BF_10_ = 1.013 x 10^-5^). The final model (Table C1) therefore included a random by-participant intercepts and main effects of Compatibility and Biological Status.

**Table C1.** Final model of error rates (ERs) in percentages using a binomial distribution and logit link function.

| Fixed Effect | Estimate | *SE* | t-value | p-value | BF_10_ |
| --- | --- | --- | --- | --- | --- |
| **(Intercept)** | **-2.699** | **0.182** | **-14.769** | **< 2 x 10^-16^***** |  |
| **Congruency** | **0.310** | **0.083** | **3.743** | **0.0002***** | **11.588** |
| **Condition** | **-0.229** | **0.082** | **-2.774** | **0.006**** | **0.522** |
| *Note:* SOA = Stimulus-Onset Asynchrony. Baseline conditions: compatible, biological status, SOA1. * *p*<.05, ***p*<.01, ***p<.001. | | | | | |

*2AFC analyses*

Performance in the two-alternative forced-choice (2AFC) task was analysed using GLMMs to assess the effect of biological status (human vs computer-generated), syllable (ba vs. da) and their interactions on by-trial accuracy. A binomial family distribution and logit link function were employed. We used the same forward model building strategy as for the SRC data. Starting with a model with random by-participant intercepts, including the effect of Biological Status significantly improved model fit (χ^2^(1)=17.862, *p*=2.375x10^-5^, BF_10_=205.408), reflecting the higher accuracy for computer-generated (*M*=6.94%, *SD*=3.52%) than the human (*M*=0.47%, *SE*=12.13%) stimuli. Adding the main effect of Syllable improved model fit (χ^2^(1)=4.116, *p*=0.042, BF_10_=0.212248), with higher ERs when responding to /da/ stimuli (*M*=2.73%, *SD*=5.70%) than to /ba/ stimuli (*M*=1.25%, *SD*=4.88%). Note that the low BF_10_ suggests that evidence for the main effect of Syllable is weak. Including the interaction between Biological Status and Syllable did not improve model fit (χ^2^(1)=0.016, *p*=0.899, BF_10_=0.027) and hence was not included in the final model.

**Table C2.** Final model of errors using a binomial distribution and logit link function for the two-alternative forced-choice (2AFC) phonetic identification task.

| Fixed Effect | Estimate | *SE* | t-value | p-value | BF_10_ |
| --- | --- | --- | --- | --- | --- |
| **(Intercept)** | **-6.121** | **0.693** | **8.836** | **<2x10^-16^** |  |
| **Biological Status** | **2.086** | **0.618** | **-3.375** | **0.0007**** | **205.408** |
| Syllable | 0.845 | 0.433 | -1.951 | 0.051 | 43.598 |
| *Note:*  Baseline conditions: human, /da/. * *p*<.05, ***p*<.01, ***p<.001. | | | | | |

**Appendix** **D**: Supplementary Analyses – Block effects.

We conducted a repeated measures four-way ANOVA using the aov_ez() function from the afex package in (Singmann et al., 2015) to assess reaction times (RTs) and compatibility effects over time. We chose this method as GLMMs failed to converge when including the main effect of and interactions including Block. Raw RTs (Schramm & Rouder, 2019) were evaluated as a function of Compatibility (compatible vs. incompatible), Biological Status (human vs. synthetic), SOA (SOA1 vs. SOA2) and Block (1 vs. 2 vs. 3 vs. 4 vs. 5). The ANOVA summary table is displayed in Table A1. The only significant main effects were that of Compatibility, Biological Status and SOA. Significant interactions occurred between Compatibility and SOA, and between Block and SOA. Follow-up contrast tests using the Bonferroni-Holm correction revealed that the effect of SOA (i.e., faster RTs at SOA2 than SOA1) was only significant in the later blocks 3 (*t*(31)=-2.939, *p*=0.019), 4 (*t*(31)=-3.569, *p*=0.005) and 5 (*t*(31)=-4.822 *p*=0.0002), but not in blocks 1 (*t*(31)=-1.269, *p*=0.428) and 2 (*t*(31)=-0.314, *p*=0.756). Crucially, no main effect or other interactions including Block reached significance.

**Table D1.** Summary of the repeated measures ANOVA aimed to assess the effect of Block on reaction times (RTs) and compatibility effects

| Main effects and interactions | *df* | *F* | *p* |
| --- | --- | --- | --- |
| **Compatibility** | **(1,31)** | **19.10** | **<.001***** |
| **SOA** | **(1,31)** | **11.16** | **.002**** |
| Biological Status | (1,31) | 0.92 | .346 |
| Block | (3.51, 108.64) | 1.86 | .131 |
| **Compatibility x SOA** | **(1,31)** | **6.67** | **.015*** |
| Compatibility x Biological Status | (1,31) | 1.78 | .192 |
| Biological Status x SOA | (1,31) | 1.02 | .321 |
| Compatibility x Block | (3.47,107.69) | 0.51 | .700 |
| **Block x SOA** | **(3.04, 94.33)** | **3.29** | **.023*** |
| Biological Status x Block | (2.67, 82.72) | 0.17 | .897 |
| Compatibility x SOA x Biological Status | **(1,31)** | 0.54 | .466 |
| Compatibility x SOA x Block | (3.42, 106.11) | 0.69 | .581 |
| Compatibility x Biological Status x Block | (3.46, 106.11) | 1.23 | .304 |
| SOA x Biological Status x Block | (3.58, 110.88) | 1.53 | .204 |
| Compatibility x SOA x Biological Status x Block | (3.57, 110.77) | 0.43 | .764 |

Bold = significant effects and interactions. * *p*<.05, ***p*<.01, ***p<.001.

**Figure D1**. Mean automatic imitation effect (incompatible RTs – compatible RTs) in milliseconds (ms) in the stimulus-response compatibility (SRC) tasks for each condition for each block. Points in the background show the raw mean AIE for each participant (points are offset on the x-axis for clarity). The boxplots indicate the first, second (median) and third quartiles, and whiskers indicate 1.5 times the interquartile range of the distribution. Black points in the foreground show the mean and error bars indicate standard errors. The lines show the line graphs of the compatible (light grey) and incompatible (dark grey) RTs plus 1 SE.

REFERENCES

Schramm, P., & Rouder, J. (2019). *Are reaction time transformations really beneficial?* <https://doi.org/10.31234/osf.io/9ksa6>

Singmann, H., Bolker, B., Westfall, J., Aust, F., & Ben-Shachar, M. S. (2015). afex: Analysis of factorial experiments. *R Package Version 0.13–145*.
